# The DNA damage response in viral-induced cellular transformation

**DOI:** 10.1038/bjc.2011.612

**Published:** 2012-01-12

**Authors:** P A Nikitin, M A Luftig

**Affiliations:** 1 Department of Molecular Genetics and Microbiology, Center for Virology, Duke University Medical Center, 213 Research Dr., CARL 424, DUMC 3054, Durham, NC 27710, USA

**Keywords:** DNA damage response, tumour virus, checkpoint

## Abstract

The DNA damage response (DDR) has emerged as a critical tumour suppressor pathway responding to cellular DNA replicative stress downstream of aberrant oncogene over-expression. Recent studies have now implicated the DDR as a sensor of oncogenic virus infection. In this review, we discuss the mechanisms by which tumour viruses activate and also suppress the host DDR. The mechanism of tumour virus induction of the DDR is intrinsically linked to the need for these viruses to promote an S-phase environment to replicate their nucleic acid during infection. However, inappropriate expression of viral oncoproteins can also activate the DDR through various mechanisms including replicative stress, direct interaction with DDR components and induction of reactive oxygen species. Given the growth-suppressive consequences of activating the DDR, tumour viruses have also evolved mechanisms to attenuate these pathways. Aberrant expression of viral oncoproteins may therefore promote tumourigenesis through increased somatic mutation and aneuploidy due to DDR inactivation. This review will focus on the interplay between oncogenic viruses and the DDR with respect to cellular checkpoint control and transformation.

Innate tumour suppression in response to oncogenic stress includes the well-characterised ARF-mediated activation and stabilisation of p53 (Zindy *et al*, 2003; Christophorou *et al*, 2006; Efeyan *et al*, 2006) and the cellular DNA damage response (DDR) that is activated following oncogene-induced replicative stress (Halazonetis *et al*, 2008). As first recognised by Halazonetis, Bartek and colleagues, tumour cells often display an activated DDR as evidenced by foci of DDR signaling proteins such as 53BP1 and activated ATM (DiTullio *et al*, 2002). This work led to the landmark papers by Bartkova *et al* (2005) and Gorgoulis *et al* (2005), which demonstrated that acute over-expression of oncogenes caused replicative stress that was sensed by the ATR signaling pathway as well as double-stranded breaks recognised by the ATM pathway. Not long after the initial characterisation of these pathways, the functional significance of the DDR activation was revealed by genetic studies indicating that ATM and Chk2 were critical tumour suppressors downstream of oncogenes including H-RasV12, Mos, Cdc6, and cyclin E (Bartkova *et al*, 2006; Di Micco *et al*, 2006). Mechanistically, these data linked the well-studied DDR signalling pathway and known tumour suppressor functions of its components, including activation of checkpoints and p53-mediated apoptosis and senescence, to an oncogene-induced replicative stress. Although much of the initial work on the DDR pathway in tumour suppression has focused on cellular oncogenes, a recent body of literature indicates that viral oncogenes also engage the DDR.

Approximately, 20% of all cases of human cancer have an infectious aetiology, with ∼80% of those being viral (Bouvard *et al*, 2009) (Table 1). For example, Epstein-Barr virus (EBV) is nearly uniformly present in African Burkitt's lymphoma and AIDS-associated non-Hodgkin's lymphomas (Rickinson and Kieff, 2006). Kaposi's sarcoma-associated herpesvirus (KSHV) is responsible for Kaposi's sarcoma (KS) and primary effusion lymphomas commonly diagnosed in AIDS patients (Mesri *et al*, 2010). Furthermore, human papillomavirus (HPV) infection is central to the development of cervical cancer and is a major contributor to the global cancer burden (Moody and Laimins, 2010). Recently, several studies have revealed the tumour-suppressive role of the DDR in response to viral oncoproteins. A unique aspect of these interactions is the interplay between the virus and the host with respect to virus replication *vs* aberrant induction of growth control genes and inhibition of apoptosis. This review will focus on complex interactions between tumour viruses and the host DDR and outcomes that promote or prevent virus-induced tumourigenesis.

## Viral oncoproteins provoke a tumour-suppressive DDR

The replication of tumour viruses is intrinsically linked to their ability to drive cell proliferation. Most of these viruses infect quiescent cells driving re-entry into the cell cycle to promote an environment conducive for viral nucleic acid replication. The consequences of such aberrant induction of cell proliferation include increased replicative stress, similar to that of cellular oncogene activation, leading to induction of the DDR. However, direct viral oncoprotein activation of the DDR also occurs through multiple mechanisms discussed below.

### Tumour viruses activate the DDR by inducing cellular hyper-proliferation

Small DNA tumour viruses antagonise the transcriptionally repressive Rb family of proteins to promote E2F-driven cellular proliferation. Uncontrolled E2F activity has been shown to activate an ATM-dependant growth-suppressive DDR (Powers *et al*, 2004; Rogoff *et al*, 2004). HPV E7 and SV40 large T antigen are classic examples of viral oncoproteins targeting Rb by direct disruption of the interaction with E2F thereby increasing S-phase promoting E2F family members to drive cellular DNA replication (DeCaprio *et al*, 1988; Dyson *et al*, 1989; Munger *et al*, 1989; Cheng *et al*, 1995; Zalvide and DeCaprio, 1995). In recent work, E6 and E7 over-expression has been shown to induce replicative stress in primary keratinocytes suggesting that potent loss of growth control through E7-mediated Rb antagonism drives uncontrolled origin firing leading to damaged DNA that may contribute to cervical cancer pathogenesis (Bester *et al*, 2011). Similarly, SV40 large T antigen is sufficient to activate an ATM-induced DDR (Boichuk *et al*, 2010). However, as described later, SV40 large T antigen activates the DDR through multiple mechanisms including those independent of Rb interaction (Boichuk *et al*, 2010). Although the polyomavirus SV40 does not cause human cancer, the recently described Merkel cell polyomavirus (MCV or MCPyV) has been found clonally integrated in Merkel cell carcinomas (MCC) (Feng *et al*, 2008) and expresses a truncated large T antigen in these tumours lacking the capacity to replicate viral DNA (Shuda *et al*, 2008). These mutant large T antigens still retain the ability to perturb cell growth through Rb antagonism and likely activate the DDR providing selective pressure for mutations in DDR genes and downstream signalling leading to MCC.

**Figure 1:**
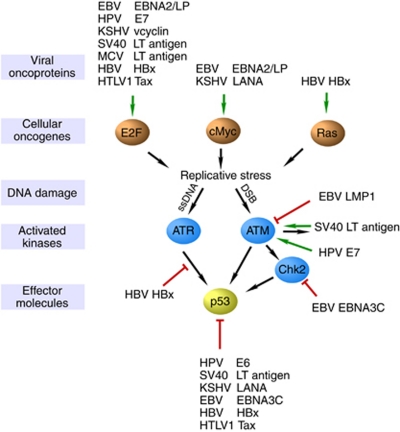
Interplay between viral oncoproteins and the host DDR. Viral oncoproteins activate cellular oncogenes (green arrows top level) in order to enter or re-enter the cell cycle, thereby inducing replicative stress and causing DNA single-stranded breaks (ssDNA). ssDNA and DNA double-stranded breaks (DSB) generated during repair of single-stranded DNA recognised by ATR and ATM kinases, respectively, which master regulate downstream signalling (all targets not shown), including activation of Chk2 and p53. Tumour virus oncoproteins modulate the function of DDR components by activating (green arrows) or suppressing (shown in red) their expression or activity.

EBV infection of human B cells *in vitro* transiently activates an ATM-dependant DDR. EBV immortalises primary human B cells in culture mimicking physiological activation and survival signals, which when constitutively active is capable of driving B-cell lymphomas *in vivo* in the immune-suppressed. Recent work on EBV-infected primary human B cells indicates that early latent oncoprotein expression drives cellular hyperproliferation and activates ATM and downstream DDR checkpoints (Nikitin *et al*, 2010). Interestingly, inhibition of ATM and its downstream kinase Chk2 early in infection markedly increases the efficiency of transformation suggesting that the DDR blocks early events in EBV-mediated B-cell outgrowth. DDR activation by EBV correlates with heightened activity of the major viral *trans*-activator EBNA2 as measured by expression of EBNA2-dependant targets such as c-Myc and CD23. Both EBNA2 activity and DDR activation wane through infected cell divisions as EBNA2 transcriptional repressors, including EBNA3C, are activated. The genetic loss of EBNA3C, in fact, promotes an uncontrolled period of hyperproliferation that induces high-level ATM activation (Nikitin *et al*, 2010). Therefore, EBV has evolved to provoke a DDR owing to its need to drive B-cell proliferation, which is then limited by full expression of its latent viral oncoproteins in immortalised lymphoblastoid cell lines (Figure 1).

Studies of the related *γ*-herpesvirus, KSHV, identified similar perturbations of the DDR signalling pathway. KSHV infects B cells and endothelial cells and can promote the development of primary effusion lymphomas and KS, two common cancers in AIDS patients (Mesri *et al*, 2010). Although the study of early events in KSHV *de novo* infection of primary cells has been limited, KSHV infection of immortalised endothelial cells *in vitro* induces the ATM signalling pathway (Koopal *et al*, 2007). Indeed, expression of the KSHV latent viral cyclin D homologue (v-cyclin) alone activates ATM. Moreover, investigation of KS tumours revealed activation of the DDR in early (patch), but not late (nodular), KS lesions (Koopal *et al*, 2007). Similar to EBV, elevated levels of DDR marks are likely induced by robust cellular proliferation. However, the downregulation of the DDR in advanced KS tumours is likely due to selection for mutations in the pathway allowing tumour cell survival.

Hepatitis B virus (HBV), which causes acute and chronic liver diseases, including cirrhosis and hepatocellular carcinoma, promotes cellular proliferation and the DDR through the pleiotropic oncoprotein HBx. Heterologous expression of HBx increases cytosolic Ca^2+^ levels leading to activation of Pyk2 and c-Src kinases (Klein and Schneider, 1997) and, ultimately, activation of Ras/Raf/MEK/ERK pathways. HBx expression can also promote p38MAPK pathway activation which upregulates E2F-dependant gene expression (Wang *et al*, 2008). Constitutive activation of these signalling pathways leads to activation of the ATR arm of the DDR pathway (Wang *et al*, 2008). The consequences of this activation, such as induction of S-phase arrest, are actually beneficial for virus replication despite being tumour suppressive (Zheng *et al*, 2011).

### Direct viral protein activation of the ATM/Chk2 signaling pathway

Beyond the growth suppressive functions of the DDR, DNA repair and activation of checkpoints may be beneficial for the replication of tumour viral genomes. Oncogenic viruses have therefore developed mechanisms to activate specific components of the DDR pathway, while strictly preventing downstream induction of apoptosis. Recent work indicates that SV40 large T antigen can serve as both a substrate for the ATM kinase as well as its direct upstream activator through binding the Nbs1 component of the ATM-activating Mre11/Rad50/Nbs1 complex (Wu *et al*, 2004; Boichuk *et al*, 2010). ATM activation is actually necessary for viral DNA replication (Zhao *et al*, 2008b). However, as discussed below, the growth-suppressive consequences of ATM activation are attenuated downstream by large T antigen enabling SV40-infected cell survival.

HPV-infected cells display increased, but non-canonical ATM pathway activation. In particular, HPV oncoprotein-expressing undifferentiated keratinocytes display an activated DDR characterised by ATM, Chk1, Chk2, and H2AX phosphorylation (Moody and Laimins, 2009). However, upon differentiation of these cells, which increases virion genome replication, an additional set of ATM targets is phosphorylated including Nbs1 (Moody and Laimins, 2009). Interestingly, E7 was demonstrated to associate with the activated Ser1981-phoshorylated form of ATM independent of differentiation or other viral proteins (Moody and Laimins, 2009). Therefore, direct association between E7 and phospho-ATM, HPV episome amplification, and viral-induced replicative stress are all capable of activating the DDR and it remains unclear which of these activities is critical in regulating HPV pathogenesis (Moody and Laimins, 2009).

### DDR activation through viral oncoprotein-mediated mitotic effects

Tumour viruses perturb normal cell cycle control in order to establish a constitutive S phase-like environment in which cellular factors are present required for viral replication. One consequence of this constitutive S-phase induction is inappropriate entry into mitosis, which activates DDR checkpoints including those triggered by Chk2 (Sato *et al*, 2010; Stolz *et al*, 2010). It was previously shown that KSHV v-cyclin expression promotes polyploidy and cytokinesis defects (Verschuren *et al*, 2002) and was subsequently confirmed by Ojala and colleagues that v-cyclin expression promotes amplification of centrosomes and intra-S-phase growth arrest (Koopal *et al*, 2007). Moreover, chemical inhibition of ATM/Chk2 led to aberrant mitoses and mitotic catastrophe in v-cyclin-expressing cells (Koopal *et al*, 2007).

In order to successfully transform cells, SV40 large T antigen targets the spindle assembly checkpoint component Bub1 leading to ATM/ATR activation (Cotsiki *et al*, 2004; Hein *et al*, 2009). Similarly, the high-risk HPV16 E6 and E7 proteins have been well documented to increase genomic instability by deregulating mitosis through the induction of multipolar spindles and centrosome duplication (Duensing *et al*, 2000). Specifically, E7 binding to nuclear mitotic apparatus protein 1 appears to deregulate normal chromosome alignment during prometaphase (Nguyen and Munger, 2009). More recently, E7 was observed to upregulate Polo-like kinase 4 (PLK4) expression leading to centriole multiplication (Korzeniewski *et al*, 2011). Therefore, multiple viral oncoproteins perturb mitosis through diverse mechanisms leading to an activated DDR.

### Tumour viruses activate the DDR through induction of reactive oxygen species (ROS)

Elevated levels of reactive oxygen species can activate DDR pathways and may result in mutagenesis during oncogenic virus infection promoting tumourigenesis. Several tumour virus oncoproteins have been shown to increase ROS levels. For example, HTLV-1 Tax expression in fibroblasts or T cells induced a ROS-dependant DDR, although the mechanism by which ROS was induced remains unknown (Kinjo *et al*, 2010). Recently, Masucci and colleagues found that the EBV protein EBNA1 induced ROS levels and consequently ATM-dependant DDR activation and ultimately chromosomal aberrations (Gruhne *et al*, 2009a). Interestingly, EBNA1 induced ROS through upregulation of the mRNA encoding the catalytic subunit of the leukocyte NADPH oxidase NOX2, which directly promotes ROS accumulation (Gruhne *et al*, 2009a). A more recent study suggests that this EBNA1-driven ROS accumulation may promote telomere dysfunction, another known molecular signal for DDR activation (Kamranvar and Masucci, 2011). Given that EBNA1 is expressed in all EBV-positive tumour cells, its ability to induce ROS may promote tumourigenesis.

## Viral proteins suppress the DDR to promote tumourigenesis

With the explicit purpose of providing an environment for virus replication, several tumour virus oncoproteins mitigate the growth-suppressive function of the DDR through altering downstream signalling events. However, the consequences of suppressing the DDR include aneuploidy and increased mutagenesis, which are major drivers of tumourigenesis. Tumour viruses have been well characterised to antagonise the function of the p53 tumour suppressor and more recently several viruses have been shown to target upstream checkpoint kinases as well.

### Tumour virus suppression of downstream DDR signalling components

The small DNA tumour viruses SV40 and HPV have been well characterised for their ability to transform cells through perturbing activation of the DDR downstream target p53 (Kress *et al*, 1979; Lane and Crawford, 1979; Scheffner *et al*, 1990, 1993). This activity is thought to be a requirement for cell survival following aberrant S phase induction due to Rb antagonism by T Ag and E7 as described above. Although large DNA tumour viruses generally do not directly promote p53 degradation or abolish its function, the KSHV latent protein LANA and EBV latent protein EBNA3C have been shown to modulate p53 activity through direct association (Friborg *et al*, 1999; Yi *et al*, 2009; Chen *et al*, 2010). Other tumour viruses also directly antagonise p53 function including the HBV oncoprotein HBx, which both inhibits p53 DNA-binding activity and sequesters p53 in the cytoplasm thereby suppressing apoptosis (Wang *et al*, 1994, 1995; Elmore *et al*, 1997; Takada *et al*, 1997). HTLV-1 Tax suppresses p53 by directly antagonising its *trans*-activating function through both NF*κ*B-dependant and NF*κ*B-independent pathways (Ariumi *et al*, 2000; Pise-Masison *et al*, 2000; Miyazato *et al*, 2005). Although many tumour viral oncoproteins have been shown to associate with p53, the extents to which these activities contribute to pathogenesis remain unclear.

### Viral oncoproteins directly target DDR checkpoint kinases

Upstream of p53 and cell cycle checkpoints are a series of DNA damage-sensing and signal-relaying kinases (Figure 1). Several viral oncoproteins directly target these upstream kinases through a number of mechanisms ultimately attenuating their function. For example, the HTLV-1 Tax oncoprotein directly binds to and inhibits signalling downstream of both Chk1 and Chk2 checkpoint kinases (Park *et al*, 2004, 2006; Gupta *et al*, 2007) as well as the upstream DNA damage-sensing DNA-PK (Durkin *et al*, 2008). Interestingly, Tax was also demonstrated to sequester the DDR components MDC1, DNA-PK and BRCA1 at artificial Tax-induced foci of pseudo-DNA damage as a unique mechanism to perturb endogenous DDR signalling pathways (Belgnaoui *et al*, 2010). Not unexpectedly, Tax expression attenuated ATM-downstream signalling leading to faster release of the G1/S checkpoint in response to ionising radiation (Chandhasin *et al*, 2008).

EBV attenuates DDR activity through indirect and also possibly direct mechanisms. As described above, at an early stage during EBV infection of primary B cells, an EBNA2-promoted, c-Myc-driven period of cellular hyperproliferation activates the DDR. Through subsequent cell divisions, the viral EBNA3C protein is expressed, which attenuates EBNA2 activity, hyperproliferation, and ultimately DDR activation (Nikitin *et al*, 2010). Therefore, as EBV-immortalised cells grow out *in vitro*, the DDR is no longer activated.

Under circumstances where EBV oncoproteins are aberrantly expressed, as evidenced in heterologous expression studies in EBV-negative B cells, DDR pathways can be directly attenuated. Specifically, Robertson and colleagues have observed a direct interaction between EBNA3C and Chk2 leading to decreased Chk2 activity, which may also contribute to DDR attenuation during primary B cell outgrowth (Choudhuri *et al*, 2007). Another study identified the latent membrane protein LMP1 as an inhibitor of ATM signalling due to transcriptional down-regulation of ATM upon LMP1 over-expression (Gruhne *et al*, 2009b). Under certain circumstances, such as in Hodgkin's lymphoma or nasopharyngeal carcinoma where LMP1 is expressed at high levels and may be important for cell survival, this activity may contribute to tumourigenesis due to the inability of ATM to trigger checkpoints and mediate efficient DNA repair.

### Viral oncoproteins perturb mitotic checkpoint signalling

Mitotic checkpoints are often provoked by viral oncoprotein promotion of cell cycle progression. Therefore, in order for these viruses to replicate in the infected cell, signalling downstream of the G2/M checkpoint must be attenuated. Several oncogenic viruses encode proteins that precisely target this checkpoint with potentially catastrophic consequences on the karyotype of surviving cells. HTLV-1 Tax expression abolishes cellular mitotic checkpoints by directly targeting and prematurely activating the anaphase-promoting complex (Liu *et al*, 2005), as well as suppressing the spindle assembly checkpoint protein Mad 1 (Jin *et al*, 1998) resulting in highly aneuploid ATL cells. Similarly, the EBV EBNA3 proteins are capable of inhibiting the canonical G2/M checkpoint through suppression of p27 levels or activity depending on the cell type (Parker *et al*, 2000; Wade and Allday, 2000; Knight and Robertson, 2004). In addition, EBNA3C is capable of suppressing the effects of mitotic poisons in part through decreasing the levels of the spindle assembly checkpoint protein BubR1 (Leao *et al*, 2007; Gruhne *et al*, 2009b). The consequence of bypassing the mitotic checkpoint and DDR signalling downstream is the accumulation of aneuploid cells that can promote tumourigenesis through copy number amplification of oncogenes or loss of tumour suppressors.

## Conclusions

In order to propagate their genomes, human tumour viruses induce robust cellular DNA replication that can lead to a replicative stress-activated host tumour-suppressive DDR. Although replicative stress is a common consequence of tumour virus infection, different strategies are used by these viruses to overcome the host DDR. Small DNA tumour viruses drive cellular DNA replication activating the host DDR, but also encode inhibitors of DDR downstream effectors such as HPV E6 or polyomavirus large T antigens that suppress p53. Activation of the host DDR induces S-phase arrest and allows such viruses to replicate in an S-phase like environment with the proper milieu of cellular replication factors. However, as p53 is a merging point of several tumour-suppressive pathways, its inhibition by viral proteins enables these viruses to avoid induction of p53-induced apoptosis or senescence. Inadvertent integration of viral genomes generates cells that produce DDR-provoking oncoproteins in the absence of viral replication. For example, MCV integrants express a truncated T antigen capable of Rb association, but not viral DNA replication (Feng *et al*, 2008). The consequences of such a scenario is the rapid proliferation of pre-malignant cells within which mutations in DDR components can be selected or, if still expressed, viral proteins can promote genomic instability by continuously inhibiting checkpoint function. It is these cells, which are far from the intentional output of the initial virus infection, that drive tumourigenesis.

The interplay between large DNA tumour viruses, such as EBV and KSHV, with the DDR is somewhat different. These viruses can replicate their episomes during the proliferation of latently infected host cells and only under specific circumstances (i.e. host cell differentiation) do they replicate genomes lytically to produce progeny virions. In order to ensure an environment that allows latent viral episome replication, viral oncoproteins promote S phase and cell proliferation. Similar to small DNA tumour viruses, these activities can lead to replicative stress and DDR activation. However, it is likely that with more complex genomes, the large DNA tumour viruses are able to attenuate the amount of replicative stress generated to maintain a successful latent infection. Such is the case for EBV, where an initial DDR-activating burst of proliferation is driven by the viral proteins EBNA2/EBNA-LP. Subsequently, expression of the EBNA3C protein attenuates EBNA2 transcriptional activity and DDR activation enabling long-term latency establishment (Nikitin *et al*, 2010). It is only in the inadvertent case when mutations in DDR components are generated by replicative stress and coupled with aberrantly over-expressed viral proteins such as EBV EBNA1 or EBNA3C or in the case of KSHV LANA or v-cyclin that these viruses might drive genomic instability and tumourigenesis. Importantly, this is not productive for the virus, but is rather a by-product of a highly evolved set of mechanisms aimed to drive cell proliferation enabling virus replication.

It is also worth noting that during lytic viral DNA replication, the nuclear sites of viral DNA synthesis often recruit DDR factors and also activate the DDR. These factors can either be beneficial for replication as is the case for ATM signalling in *γ*-herpesviruses (Tarakanova *et al*, 2007; Bouvard *et al*, 2009; Li *et al*, 2011) and papillomaviruses (Moody and Laimins, 2009) or detrimental as in the case of the ATM-activating Mre11/Rad50/Nbs1 proteins precluding processing of adenovirus DNA ends before packaging (Stracker *et al*, 2002). Therefore, the DDR can serve quite distinct functions depending on the molecular nature of the DNA damage and need for recombination and repair during genome replication.

In summary, the DDR can be activated directly by aberrant expression of oncoproteins, cellular or viral, or as a consequence of cellular proliferation-induced replicative stress. DNA tumour virus-driven cellular transformation occurs as a by-product of the virus promoting the cell cycle in order to establish an appropriate environment with the requisite DNA replication machinery and repair factors necessary for viral DNA replication. Similarly, viruses such as HTLV-1 must activate the infected T-cell in order to promote a favourable environment for proviral DNA integration. However, in the inadvertent setting such as following aberrant integration of viral genomes where loss of normal viral replication function occurs or other changes lead to increased viral oncoprotein expression, a constitutively activated DDR is triggered. DDR signalling typically limits viral oncogenesis, but also provides selective pressure for mutations in DDR signalling components that promote tumourigenesis. The delicate balance between virus replication, latency, and the extent of activation of the DDR ultimately dictates whether an infected cell will give rise to a productive cycle generating progeny virions or a tumour.

## Figures and Tables

**Table 1:** Human oncogenic viruses and their interactions with the host DNA damage response

**Oncogenic virus**	**Tumors associated with virus infection**	**Oncoproteins involved in DDR**	**References**
EBV	Burkitt's lymphoma, post-transplant lymphoma, non-Hodgkin's/diffuse large B cell lymphomas, nasopharyngeal carcinoma, gastric carcinoma	EBNA2/LP → DDR	(Nikitin *et al*, 2010)
		EBNA1 → ROS → DDR	(Gruhne *et al*, 2009a)
		EBNA3C ⊣ early DDR	(Nikitin *et al*, 2010)
		EBNA3C⊣Chk2,p53	(Choudhuri *et al*, 2007; Yi *et al*, 2009)
		EBNA3C⊣ G2/M checkpoint	(Gruhne *et al*, 2009b; Parker *et al*, 2000)
		LMP1⊣ATM	(Gruhne *et al*, 2009b)
KSHV	Kaposi's sarcoma, primary effusion lymphoma	v-cyclin → ATM	(Koopal *et al*, 2007)
		LANA → myc → DDR	(Liu *et al*, 2007)
		LANA:p53	(Chen *et al*, 2010; Friborg *et al*, 1999)
HPV	Cervical cancer, ovarian cancer	E6,E7 → repl stress → DDR	(Bester *et al*, 2011)
		E7:pATM	(Moody and Laimins, 2009)
		E6⊣p53	(Scheffner *et al*, 1990)
HBV	Hepatocellular carcinoma	HBV → ATR	(Wang *et al*, 2008; Zhao *et al*, 2008*a*)
		HBx → Ras → DDR	(Klein and Schneider, 1997)
		HBx ⊣ p53	(Wang *et al*, 1994)
HTLV I	ATL	Tax⊣DNA-PK	(Durkin *et al*, 2008)
		Tax⊣Chk1/Chk2	(Park *et al*, 2004; Park *et al*, 2006)
		Tax⊣p53	(Ariumi *et al*, 2000; Pise-Masison *et al*, 2000)

Abbreviations: ATL=adult T-cell leukaemia; DDR=DNA damage response; EBV=Epstein–Barr virus; HBV=hepatitis B virus; HPV=human papillomavirus; HTLV-I=human T-lymphotropic virus type I.
